# Basic chloroplast genome characterization of *Phalaenopsis stobartiana* (Orchidaceae) from China

**DOI:** 10.1080/23802359.2022.2026831

**Published:** 2022-01-24

**Authors:** Ying-Hui Cao, Mei-Juan Hu, Yan Tong, Yan-Ping Zhang, Rui-Yue Zheng, Kai Zhao, Dong-Hui Peng, Yu-Zhen Zhou

**Affiliations:** a College of Landscape Architecture, Fujian Agriculture and Forestry University, Fuzhou, China; b College of Life Sciences, Fujian Normal University, Fuzhou, China

**Keywords:** *Phalaenopsis stobartiana*, chloroplast genome, phylogenetic analysis

## Abstract

*Phalaenopsis stobartiana* Reichenbach f. 1877 is mainly distributed in Yunnan province of China and has a high ornamental and breeding value. Here, we reported the chloroplast genome of *P. stobartiana*. The length of the chloroplast genome was 145,900 bp, encoding 120 genes. The average GC content was 36.8%. Phylogenetic analysis revealed that *P. stobartiana* and *P. wilsonii* are closely related. The chloroplast genome could be used for further phylogenetic research, and provide molecular data for future genetic protection and breeding programs.

Phalaenopsis is a popular ornamental flower, commonly known as moth orchids, their blooms last for months. More than 70 origin species within the *Phalaenopsis* genus and many available cultivars. *Phalaenopsis stobartiana* is a miniature epiphytic orchid, growing on trunks of trees in dense forests, distributed at 1300 − 1400 m altitude in the Yunnan province, China (Chen and Jeffrey 2009). It presents a high ornamental and breeding value, exhibiting green sepals and a distinctive scent (Christenson 2001). Unfortunately, with the ruthless collection and habitat loss, *P. stobartiana* has been listed as the first class protected wild plant in China (http://www.iplant.cn/rep/prot/Phalaenopsis%20stobartiana). In addition, the classification of *Phalaenopsis* is still confusing due to limited samples species coverage and genetic resources (Deng et al. 2015). Therefore, the cp genome information of *P. stobartiana* would provide valuable molecular data for genetic protection and phylogenetic studies.

The plant samples of *P. stobartiana* were obtained from Yingjiang County, Yunnan Province, China (24.7 N, 97.9 E), and they were stored in Fujian Agriculture and Forestry University Herbarium (Voucher specimen: GL-YN2019-17A, Yuzhen Zhou, zhouyuzhencn@163.com). We used the modified CTAB method (Sahu et al. 2012) to extract the genomic DNA of *P stobartiana* from fresh leaves. The DNA was fragmented using Covaris, and the fragments of DNA between 200 and 400 bp were selected. The extracted DNA then underwent end-repairing, phosphorylation, and A-tailing reactions. Thereafter, the fragments were further amplified and circularized. Single stranded DNA circles were formatted as the final library and sequenced using pair-end 100 bp reads on BGISEQ-500. Approximately 14 GB data were generated from the BGI-500 platform and deposited in the GenBank SRA database (accession number: SRX12193139). The data were assembled using SPAdes 3.13.1 software. The assembled circular chloroplast genome was annotated and corrected using GeSeq (Tillich et al. 2017) and Geneious Prime v2020.2.1 software. After the annotation results were verified through GB2sequin (Lehwark and Greiner 2019), we submitted the chloroplast genome of *P. stobartiana* to GenBank (accession number: MW531729).

The *P stobartiana* chloroplast genome was 145,900 bp in size, with an LSC region (85,295 bp), SSC region (10,881 bp), and two IR regions (24,862 bp each). The average GC content was 36.8%. The cp genome encoded 74 protein-coding genes, 38 tRNAs, and 8 rRNAs.

To identify the *P. stobartiana’s* phylogenetic position, 26 complete chloroplast genomes from *Aeridinae* and two outgroups were aligned using MAFFT v.7. The maximum likelihood tree was constructed using RAxML-HPC2 on CIPRES with the GTRCAT model and 1000 bootstrap replicates (Stamatakis 2014). All the sequences were downloaded from NCBI GenBank. The result indicated that *P. stobartiana* was sister to *P. wilsonii* with high bootstrap support (Figure 1).

**Figure 1.**
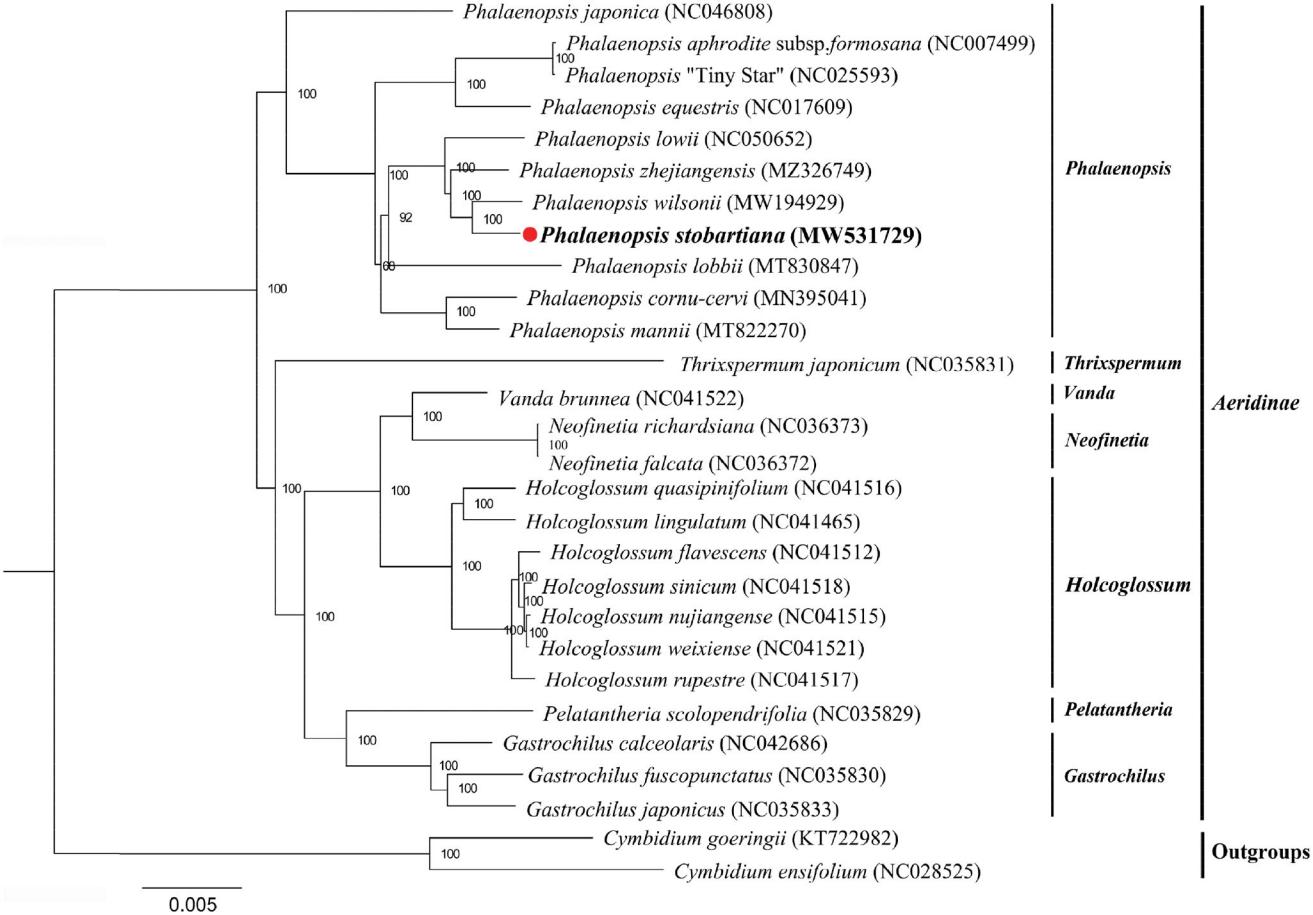
Maximum-likelihood (ML) phylogenetic tree of 26 chloroplast sequences in subtribe *Aeridinae*. *Cymbidium goeringii* and *C. ensifolium* were used as outgroups. The position of *P. stobartiana* was marked with the red circle.

## Ethical approval

The material involved in the article does not involve ethical conflicts. This study was permitted by the Key Laboratory of National Forestry and Grassland Administration for Orchid Conservation and Utilization, FAFU, China. All collection and sequencing work was strictly executed under local legislation and related laboratory regulations to protect wild resources.

## Data Availability

The plant samples were identified by Dr. Yu-Zhen Zhou, and deposited at the Fujian Agriculture and Forestry University Fuzhou, Fujian, China (Voucher specimen: GL-YN2019-17A). The genome sequence data that support the findings of this study are openly available in GenBank of NCBI at https://www.ncbi.nlm.nih.gov, reference number MW531729. The associated Bio-Sample, BioProject and SRA numbers are SAMN21439087, PRJNA763377, and SRX12193139, respectively.

## References

[CIT0001] Chen SC, Jeffrey JW. 2009. Flora of China. Vol. 25. Beijing: Sciences Press; St. Louis: Missouri Botanical Garden Press; p. 478–483.

[CIT0002] Christenson EA. 2001. Phalaenopsis: a monograph. OR, USA: Timber Press; p. 56–58.

[CIT0003] Deng H, Zhang GQ, Liu ZJ, Wang Y. 2015. A new species and a new combination of Phalaenopsis (Orchidaceae: Epidendroideae: Aeridinae): evidence from morphological and DNA analysis. Phytotaxa. 238(3):243–254.

[CIT0004] Lehwark P, Greiner S. 2019. GB2sequin – a file converter preparing custom GenBank files for database submission. Genomics. 111(4):759–761. 29842948 10.1016/j.ygeno.2018.05.003

[CIT0005] Sahu SK, Thangaraj M, Kathiresan K. 2012. DNA extraction protocol for plants with high levels of secondary metabolites and polysaccharides without using liquid nitrogen and phenol. ISRN Mol Biol. 2012:205049. 27335662 10.5402/2012/205049 PMC4890884

[CIT0006] Stamatakis A. 2014. RAxML version 8: a tool for phylogenetic analysis and post-analysis of large phylogenies. Bioinformatics. 30(9):1312–1313. 24451623 10.1093/bioinformatics/btu033 PMC3998144

[CIT0007] Tillich M, Lehwark P, Pellizzer T, Ulbricht-Jones ES, Fischer A, Bock R, Greiner S. 2017. GeSeq- versatile and accurate annotation of organelle genomes. Nucleic Acids Res. 45(W1):W6–W11. 28486635 10.1093/nar/gkx391 PMC5570176

